# Proteins Marking the Sequence of Genotoxic Signaling from Irradiated Mesenchymal Stromal Cells to CD34+ Cells

**DOI:** 10.3390/ijms22115844

**Published:** 2021-05-29

**Authors:** Vanessa Kohl, Oliver Drews, Victor Costina, Miriam Bierbaum, Ahmed Jawhar, Henning Roehl, Christel Weiss, Susanne Brendel, Helga Kleiner, Johanna Flach, Birgit Spiess, Wolfgang Seifarth, Daniel Nowak, Wolf-Karsten Hofmann, Alice Fabarius, Henning D. Popp

**Affiliations:** 1 Department of Hematology and Oncology, Medical Faculty Mannheim, Heidelberg University, 68167 Mannheim, Germany; Vanessa.Kohl@medma.uni-heidelberg.de (V.K.); Susanne.Brendel@medma.uni-heidelberg.de (S.B.); Helga.Kleiner@medma.uni-heidelberg.de (H.K.); Johanna.Flach@medma.uni-heidelberg.de (J.F.); Birgit.Spiess@medma.uni-heidelberg.de (B.S.); Wolfgang.Seifarth@medma.uni-heidelberg.de (W.S.); Daniel.Nowak@medma.uni-heidelberg.de (D.N.); w.k.hofmann@medma.uni-heidelberg.de (W.-K.H.); Alice.Fabarius@medma.uni-heidelberg.de (A.F.); 2 Department of Clinical Chemistry, University Medical Center Mannheim, 68167 Mannheim, Germany; Oliver.Drews@umm.de (O.D.); Victor.Costina@umm.de (V.C.); 3 Department of Radiation Oncology, Medical Faculty Mannheim, Heidelberg University, 68167 Mannheim, Germany; Miriam.Bierbaum@medma.uni-heidelberg.de; 4 Department of Orthopedics and Trauma Surgery, Medical Faculty Mannheim, Heidelberg University, 68167 Mannheim, Germany; Ahmed.Jawhar@medma.uni-heidelberg.de; 5 Department of Orthopedics and Trauma Surgery, Diakonissen Hospital, 68163 Mannheim, Germany; h.roehl@diako-mannheim.de; 6 Department of Medical Statistics and Biomathematics, Medical Faculty Mannheim, Heidelberg University, 68167 Mannheim, Germany; Christel.Weiss@medma.uni-heidelberg.de

**Keywords:** irradiation, genotoxic signals, non-targeted effects, mesenchymal stromal cells, CD34+ cells, myeloid neoplasms

## Abstract

Non-targeted effects (NTE) of ionizing radiation may initiate myeloid neoplasms (MN). Here, protein mediators (I) in irradiated human mesenchymal stromal cells (MSC) as the NTE source, (II) in MSC conditioned supernatant and (III) in human bone marrow CD34+ cells undergoing genotoxic NTE were investigated. Healthy sublethal irradiated MSC showed significantly increased levels of reactive oxygen species. These cells responded by increasing intracellular abundance of proteins involved in proteasomal degradation, protein translation, cytoskeleton dynamics, nucleocytoplasmic shuttling, and those with antioxidant activity. Among the increased proteins were THY1 and GNA11/14, which are signaling proteins with hitherto unknown functions in the radiation response and NTE. In the corresponding MSC conditioned medium, the three chaperones GRP78, CALR, and PDIA3 were increased. Together with GPI, these were the only four altered proteins, which were associated with the observed genotoxic NTE. Healthy CD34+ cells cultured in MSC conditioned medium suffered from more than a six-fold increase in γH2AX focal staining, indicative for DNA double-strand breaks, as well as numerical and structural chromosomal aberrations within three days. At this stage, five proteins were altered, among them IQGAP1, HMGB1, and PA2G4, which are involved in malign development. In summary, our data provide novel insights into three sequential steps of genotoxic signaling from irradiated MSC to CD34+ cells, implicating that induced NTE might initiate the development of MN.

## 1. Introduction

Ionizing radiation (IR) is associated with the generation of electrons and free radicals, which may damage DNA, proteins, lipids, and other structures [1]. Some of the detrimental effects of IR, such as the development of secondary neoplasias, might be associated with the release of danger signals based on DNA damage and apoptosis gradients between irradiated and non-irradiated cells [2]. The response in irradiated cells might be initiated by the DNA damage response, apoptosis, and inflammation [2]. Numerous protein-protein interaction networks have been identified in the signaling processes [2,3]. The macrophage system is assumed to be critically involved in these signaling processes in vivo [4,5].

Non-targeted effects (NTE) comprise systemic ‘out-of-field’ effects of IR and may contribute to malignant transformation [6]. NTE might be initiated in HSPC by nearby or distant irradiated mesenchymal stromal cells (MSC) [7,8]. NTE may emerge as DNA damage (for example gene mutations, chromosomal aberrations, micronuclei, increased γH2AX foci), cell death (for example apoptosis, necrosis) and induction of cell survival mechanisms (for example adaptive response, increased DNA repair) [9,10,11,12]. While NTE have been demonstrated in mouse HSPC [7,8], they have so far not been detected in cultured human stem cells [13].

The sequence of genotoxic signaling from irradiated cells to non-irradiated cells might be initiated in the irradiated cells by calcium fluxes [14] and mitochondrial metabolites [14,15]. Consecutively, signal transmission between irradiated and non-irradiated cells may occur by messengers such as nitric oxide (NO) [16] and reactive oxygen species (ROS) [17]. Further, inflammatory cytokines such as IL-1 beta [18] IL-8 [18] TNF-alpha [18] and TGF-beta-1 [18,19,20] may be excreted by irradiated cells. A role of gap junctions in cell-to-cell signaling from irradiated to non-irradiated cells has been described as well [21,22]. In addition, cathepsin B, a lysosomal cysteine protease, which plays an important role in intracellular proteolysis, was identified as a genotoxic signaling molecule [23]. Moreover, nucleic acids such as microRNA and mitochondrial DNA may be secreted by irradiated cells in exosomes and exosome-like vesicles and contribute to genotoxic signaling [24,25]. Further, cell-free chromatin released from dying irradiated cells may integrate into the genomes of bystander cells and cause chromosomal instability (CIN) [26]. In this last step, NO [27], ROS [27], calcium fluxes [28], regulators such as transcription factor NF-kappa-B [29] and mediators such as MAP kinases (MAPKs) [30] might be induced in the non-irradiated bystander cells.

Cells in the human body might be exposed to different irradiation sources. The average annual radiation dose per person in the U.S. is 6.2 mSv from man-made (e.g., computed tomography, nuclear medicine) and natural background sources (e.g., radon, cosmic radiation) (https://www.epa.gov/radiation/radiation-sources-and-doses, accessed on 28 May 2021). The dose may increase in patients exposed to CT scans (<30 (–50) mGy per scan) and radiation therapy (up to 60 (–80) Gy applied in fractionated doses of about 2 Gy/day). While NTE might be relevant at low doses <100 mGy, systemic NTE might be critical at high doses >1 Gy as well.

In summary, a sequence of genotoxic signaling from irradiated human MSC to HSPC might cause NTE in HSPC potentially initiating MN development. Therefore, our study was designed to investigate firstly NTE in human CD34+ cells in terms of DNA damage and CIN and secondly mediators (I) in irradiated human MSC as NTE source, (II) in cell/debris-free MSC conditioned supernatant and (III) in human bone marrow CD34+ cells by analysis of ROS levels and proteome shifts.

## 2. Results

### 2.1. ROS in MSC and CD34+ Cells

**Figure 1:**
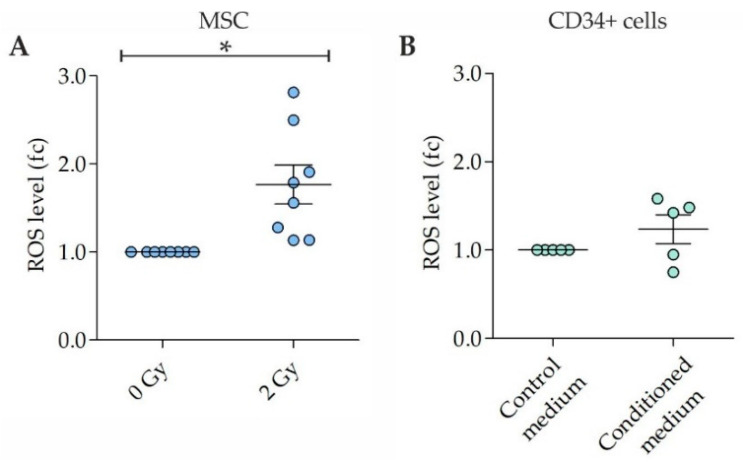
Reactive oxygen species (ROS) levels in irradiated mesenchymal stromal cells (MSC) and CD34+ cells grown in MSC conditioned medium. (**A**) ROS levels in 2 Gy-irradiated MSC at 4 h after irradiation. *n* = 8 samples. (**B**) ROS levels in CD34+ cells grown for 3 days in medium conditioned by 2 Gy-irradiated MSC. *n* = 5 samples. Data are presented as means ± SEM. fc, fold change. One sample *t*-tests. * *p* < 0.05.

ROS were analyzed in 2 Gy-irradiated MSC samples at 4 h after irradiation and in non-irradiated control MSC. Increased ROS levels were detected in irradiated MSC (fold change (fc) = 1.8 ± 0.2; mean ± SEM), when compared to non-irradiated MSC (fc = 1) (Figure 1A). Furthermore, ROS were analyzed in CD34+ cell samples expanded for 3 days in untreated medium followed by culture for 3 days in MSC conditioned medium or control medium, respectively. ROS levels were slightly increased in CD34+ cells grown in MSC conditioned medium (fc = 1.2 ± 0.2), when compared to ROS levels in CD34+ cells grown in the control medium (fc = 1) (Figure 1B).

### 2.2. DNA Damage in CD34+ Cells

**Figure 2:**
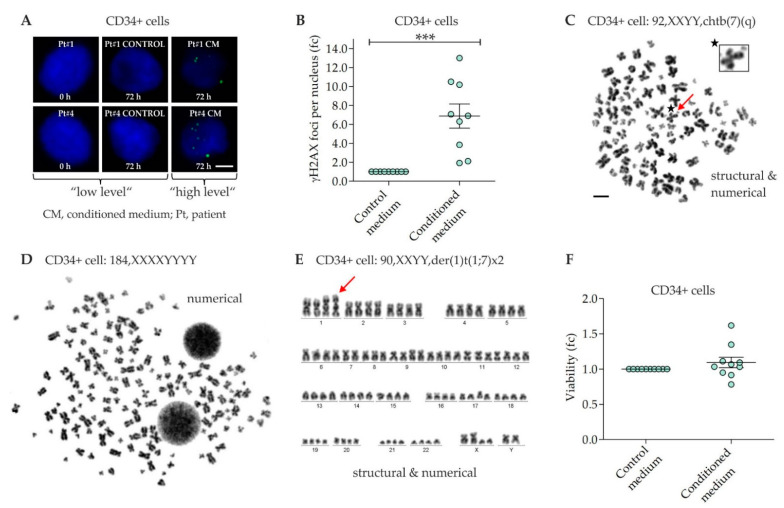
Non-targeted effects in CD34+ cells. (**A**) Exemplary immunofluorescence images of γH2AX foci (green, Alexa 488) in nuclei (blue, DAPI) of CD34+ cells grown for 3 days in medium conditioned by 2 Gy-irradiated mesenchymal stromal cells (MSC). Scale bar, 5 µm. (**B**) γH2AX foci in CD34+ cells grown for 3 days in control medium and MSC conditioned medium. *n* = 9 samples. (**C**,**D**) Exemplary aberrant metaphases of different donor CD34+ cells grown for 3 days in MSC conditioned medium. Scale bar, 10 µm. (**E**) Exemplary aberrant karyotype of a donor CD34+ cell grown for 3 days in MSC conditioned medium. (**F**) Viability of CD34+ cells grown for 3 days in control medium and MSC conditioned medium. *n* = 10 samples. Data in (**B**,**F**) are presented as means ± SEM. fc, fold change. Wilcoxon two-sample test. *** *p* < 0.005.

γH2AX foci were analyzed in CD34+ cell samples expanded for 3 days in untreated medium followed by culture for 3 days in MSC conditioned medium or control medium, respectively. γH2AX foci levels were increased in CD34+ cells grown in MSC conditioned medium (fc = 6.9 ± 1.3), when compared to γH2AX foci levels in CD34+ cells grown in control medium (fc = 1) (Figure 2A,B).

### 2.3. Chromosomal Instability in CD34+ Cells

Metaphases were analyzed in CD34+ cell samples expanded for 3 days in untreated medium followed by culture for 3 days in MSC conditioned medium or control medium, respectively (Figure 2C–E, Table 1). Structural and numerical chromosomal aberrations were detected in 50% and 92% of CD34+ cell samples grown in MSC conditioned medium, respectively, when compared to normal karyotypes detected in CD34+ cell samples grown in control medium. In particular, chromatid breaks (chtb) such as chtb(5q), chtb(6p), chtb(7q), chtb(10q), chtb(11q), and chtb(13q), translocations such as der(1)t(1;7) and aneuploidies such as tetraploidies and octoploidies, were observed in CD34+ cells grown in MSC conditioned medium.

### 2.4. Viability of CD34+ Cells

Viability was assessed in CD34+ cell samples grown for 3 days in untreated medium followed by culture for 3 days in MSC conditioned medium or control medium, respectively. The viability of CD34+ cells grown in MSC conditioned medium (fc = 1.1 ± 0.1) was similar, when compared to viability of CD34+ cells grown in control medium (fc = 1) (Figure 2F).

### 2.5. Proteome Analysis in MSC, MSC Conditioned Medium and CD34+ Cells

**Figure 3:**
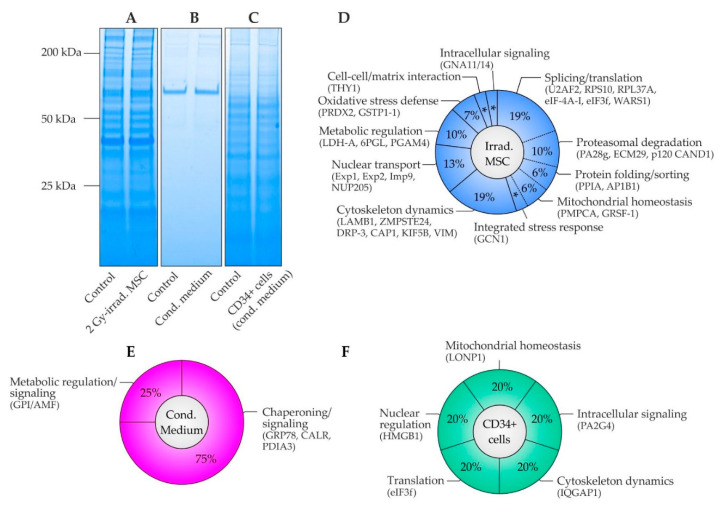
Comparative proteome analysis in mesenchymal stromal cells (MSC), MSC conditioned medium and CD34+ cells. (**A**) Exemplary SDS-PAGE of lysates of non-irradiated and 2 Gy-irradiated MSC, (**B**) control medium and medium conditioned by 2 Gy-irradiated MSC and (**C**) lysates of CD34+ cells grown in control medium and in medium conditioned by 2 Gy-irradiated MSC. (**D**) Proteome alterations in mesenchymal stromal cells (MSC) (*n* = 5 replicates, * 3%), (**E**) MSC conditioned medium (*n* = 4 replicates) and (**F**) CD34+ cells grown for 3 days in MSC conditioned medium (*n* = 5 replicates).

Comparative proteome analysis was performed in patient samples with (I) lysates of irradiated and non-irradiated MSC, (II) MSC conditioned and control medium and (III) lysates of CD34+ cells grown in MSC conditioned and control medium (Figure 3 and Table 2 and Tables S1–S3). In MSC, 31 of 1924 identified proteins (1.6%) were regulated at least two-fold within 4 h upon a single irradiation dose of 2 Gy compared to controls (Table S1). The majority of proteins demonstrated increased abundances (94%, Table 2).

About 45% of the proteins participated in protein synthesis, processing, and degradation. Increased splicing factor U2AF 65 kDa subunit (U2AF2) suggested exaggerated pre-mRNA splicing and 3′-end processing. Further, elevated 40S ribosomal protein S10 (RPS10), eukaryotic initiation factor 4A-I (eIF-4A-I), eukaryotic translation initiation factor 3 subunit F (eIF3f) and tryptophan-tRNA ligase (WARS1) indicated activation of protein synthesis in irradiated MSC. On the other hand, decreased 60S ribosomal protein L37a (RPL37A) in irradiated MSC was in line with common suppression in distinct cancers [31]. In addition, GCN1 an activator protein of eIF-2-alpha kinase/GCN2 on translating ribosomes was increased resulting in activation of transcriptional factor ATF4, which is a regulator of the integrated stress response. Further, elevated peptidyl-prolyl cis-trans isomerase A (PPIA) suggested activated protein folding in the endoplasmic reticulum (ER), as was the same for increased AP-1 complex subunit beta-1 (AP1B1) regarding protein sorting in the trans-Golgi network and/or endosomes. In addition, high abundances of mitochondrial-processing peptidase subunit alpha (PMPCA) and G-rich sequence factor 1 (GRSF-1) suggested adaption of mitochondrial protein homeostasis in irradiated MSC. Furthermore, increased levels of proteasome activator complex subunit 3 (PA28g), proteasome adapter and scaffold protein ECM29 (ECM29) and cullin-associated NEDD8-dissociated protein 1 (p120 CAND1) indicated proteasomal degradation of proteins resulting in altered protein homeostasis in irradiated MSC.

About 19% of affected proteins in irradiated MSC were part of the cytoskeleton and participated in its dynamic regulation. Abundances were increased for laminin subunit beta-1 (LAMB1), which is a component of the basal membrane, CAAX prenyl protease 1 homolog (ZMPSTE24), which forms lamin A in the nuclear lamina, dihydropyrimidinase-related protein 3 (DRP-3) and adenylyl cyclase-associated protein 1 (CAP1), which are cytoskeleton regulators, as well as kinesin-1 heavy chain (KIF5B), which is a microtubule-dependent motor protein. The only decreased cytoskeleton protein in irradiated MSC was vimentin (VIM), which plays a critical role in anchoring cell organelles, suggesting organelle repositioning in irradiated MSC.

Elevated nuclear transporter proteins accounted for about 13% of the altered proteome in irradiated MSC. Increased exportin-1 (Exp1), exportin-2 (Exp2), importin-9 (Imp9) and nuclear pore complex protein Nup205 (NUP205) suggested activation of the import and/or export of nuclear proteins. The remaining proteins accounted for about 10%, 6%, 3%, and 3% of the altered proteome in irradiated MSC and took part in metabolic regulation, oxidative stress defense, cell-cell/matrix interaction, and intracellular signaling, respectively. Increased L-lactate dehydrogenase A chain (LDH-A), which synthesizes (S)-lactate from pyruvate, 6-phosphogluconolactonase (6PGL), which is involved in the pentose phosphate pathway, and probable phosphoglycerate mutase 4 (PGAM4), which participates in glycolysis, together indicated a metabolic shift in irradiated MSC in the benefit of rapid supply with the energy carrier adenosine triphosphate (ATP) and precursors for nucleotide and amino acid biosynthesis. In addition, elevated peroxiredoxin-2 (PRDX2) and glutathione S-transferase P (GSTP1-1) pointed out activation of oxidative stress defense. Further, elevated Thy-1 membrane glycoprotein (THY1) and guanine nucleotide-binding protein subunit alpha-11/14 (GNA11/14) suggested activation of cell-cell/matrix interactions and intracellular signaling pathways. The specific role of these latter proteins in the radiation response remains elusive.

In the corresponding secretome in MSC conditioned medium, 4 of 265 identified proteins (1.5%) were found increased in their abundance by factor 2 or higher 4 h after irradiation versus controls (Table S2). Remarkably, 75% of the altered proteins were key proteins in the ER and known for their role in protein folding as well as protein quality control. In particular, ER chaperone BiP (GRP78), which is a key chaperone involved in the unfolded protein response (UPR) and ER-associated protein degradation pathway (ERAD), calreticulin (CALR), which is involved in the folding of glycoproteins in the calreticulin/calnexin cycle and in calcium homeostasis as well as protein disulfide-isomerase A3 (PDIA3), which catalyzes the rearrangement of disulfide bonds for correct folding of newly-synthesized glycoproteins, were all increased in MSC conditioned medium. Beyond their canonical function in chaperoning, they may exert non-canonical functions in oncogenic signaling. In addition, glucose-6-phosphate isomerase (GPI) was increased in MSC conditioned medium, which is identical to secreted autocrine motility factor (AMF) involved in growth/motility-mediating AMF receptor signaling.

Exposure of CD34+ cells to the MSC conditioned medium for 3 days induced quantitative changes of a minimum factor 2 in 5 of 2003 identified proteins (0.25%, Table S3). Hence, the response in CD34+ cells to MSC conditioned medium affected much less proteins than in MSC, which were directly exposed to irradiation. Similar to MSC, affected proteins participated in mitochondrial protein homeostasis, intracellular signaling, cytoskeleton dynamics, translation, and nuclear regulation. Among the differentially abundant proteins, increased mitochondrial lon protease homolog (LONP1) exerts protease and chaperone activity for regulating mitochondrial protein homeostasis. Further, elevated proliferation-associated protein 2G4 (PA2G4) functions in growth-mediating ERBB3 signaling. On the other hand, decreased Ras GTPase-activating-like protein IQGAP1 (IQGAP1) suggested reduced actin dynamics as was the same for diminished eukaryotic translation initiation factor 3 subunit F (eIF3f) regarding inactivation of the eIF-3 translation initiation complex. Finally, decreased high mobility group protein B1 (HMGB1) was detected, which is critically involved in nuclear processes such as replication, transcription, and chromatin remodeling.

## 3. Discussion

The aim of our study was to analyze NTE in human CD34+ cells and the sequence of genotoxic signaling from irradiated human MSC to CD34+ cells as a potential mechanism of MN initiation, which are termed in this context therapy-related MN (t-MN). For this purpose, NTE were analyzed in CD34+ cells grown in medium conditioned by 2 Gy-irradiated MSC. Furthermore, ROS and proteome shifts were assessed in (I) irradiated MSC, (II) MSC conditioned medium and (III) CD34+ cells exposed to MSC conditioned medium. Naturally, our data present a snap-shot in the dynamic process of the radiation response, the release of genotoxic mediators and the induction of NTE. Overall, based on radiobiological considerations, IR-induced leukemogenesis might be a function of several parameters including targeted effects, NTE, and a given HSPC predisposition specified by gene mutations interfering with processes such as genome maintenance mechanisms and the DNA damage response.

Increased numbers of γH2AX foci as well as structural and numerical chromosomal aberrations were detected in CD34+ cells grown in MSC conditioned medium, when compared to CD34+ cells grown in control medium. The increased numbers of γH2AX foci in CD34+ cells grown in MSC conditioned medium may not only indicate critical DNA damage [32] potentially contributing to MN initiation for example by activation of oncogenes or inactivation of tumor suppressor genes. In addition, γH2AX foci may indicate double-strand breaks involved in chromosomal rearrangements such as deletions, inversions, and translocations. Indeed, t-MN related chromosomal aberrations were found in CD34+ cells grown in MSC conditioned medium, when compared to whole chromosomes in CD34+ cells grown in control medium. Particularly, chtb(5q), chtb(7q), chtb(11q) and chtb(13q), which were found in CD34+ cells grown in MSC conditioned medium, coincided well with del(5q), del(7q), t(11q23.3) and del(13q), which are present in about 42%, 49%, 3%, and <5% of t-MN, respectively [33,34]. In addition, t-MN related aneuploidies, for example tetraploidies and octoploidies, were detected in CD34+ cells grown in MSC conditioned medium. Numerical chromosomal aberrations are caused by defects in mitosis such as chromosomal non-disjunction and cytokinesis failure [35]. In this way, tetraploid cells in our experiments demonstrate a kind of clonal evolution as octoploid cells only arise from dividing tetraploid cells. Moreover, tetraploidies are hallmark precursor lesions in diverse cancers such as cervical cancer and neuroblastoma, and occur in about 1% of AML but in 13% of t-AML cases [35,36]. As tetraploid cells harbor 4n centrosomes, multipolar spindles may form potentially driving a CIN phenotype. With ongoing dedifferentiation, CIN may aggravate in CD34+ cells for example by frequent inactivation of *TP53*, which may result in rapid t-MN development [35]. Overall, the increased numbers of γH2AX foci and chromosomal aberrations did not seem to affect viability of CD34+ cells within the observation period as viability was similar in CD34+ cells grown in MSC conditioned medium and in CD34+ cells grown in control medium.

ROS were analyzed in irradiated MSC and CD34+ cells grown in MSC conditioned medium for their potential participation in genotoxic signaling from irradiated MSC to CD34+ cells. Increased ROS levels were detected in irradiated MSC and in CD34+ cells grown in MSC conditioned medium. While ROS are genotoxic molecules generated by endogenous and exogenous sources in each cell, ROS may also function as important regulators of intracellular signaling pathways, for example by covalent modification of specific cysteine residues in redox-sensitive target proteins [37]. Oxidation of specific cysteine residues in turn can lead to reversible modification of enzyme activity [37] with effects on diverse pathways including metabolism, differentiation, and proliferation [38]. Hence, ROS may not only induce DNA damage but also dysregulate cellular pathways, thereby contributing to the transformation of CD34+ cells. Furthermore, ROS might be both cause and consequence of the detected proteome shifts in CD34+ cells exposed to MSC conditioned medium.

In order to identify potential mediators for the observed oncogenic transformation in CD34+ cells as well as mechanisms leading to their release in MSC and transduction in CD34+ cells, comparative proteome analyses were performed in three tiers of (I) irradiated MSC, (II) MSC conditioned medium, and (III) CD34+ cells grown in MSC conditioned medium. Among these three comparisons, irradiated MSC showed the largest change in proteome, which is in accordance with the impact of the primary stimulus. Still, the response can be regarded as rather moderate, because only 1.6% of the analyzed proteome was altered by a factor 2 or higher. An underlying mechanism might be the relative radioresistance of MSC [39]. Given that MSC survive a dose of 2 Gy, a substantial release of cell-free chromatin and its contribution to NTE in CD34+ cells can be excluded [26]. The majority of altered proteins in MSC took part in the translation, protein folding and sorting as well as protein degradation, indicating disturbed protein homeostasis and required replacement, repair, and degradation of proteins. Furthermore, differentially abundant proteins participating in cytoskeleton dynamics, nuclear transport, metabolic regulation, oxidative stress defense, cell-cell/matrix interactions, and intracellular signaling were detected in irradiated MSC.

Three of the few quantitatively altered proteins in MSC conditioned medium upon irradiation were key ER chaperones (GRP78, CALR, PDIA3) involved in protein folding and their quality control. The highest increase of the three chaperones was observed for GRP78, which dissociates from the luminal domains of IRE1, PERK and ATF6 in consequence of ER stress, resulting in activation of the UPR [40] and the ERAD pathway [41]. In turn, ERAD relies on substrate degradation through the ubiquitin-proteasome system. Notably, two proteasome activator proteins (ECM29 and PA28g) as well as a key assembly factor of SCF E3 ubiquitin ligase complexes (p120 CAND1) were all increased in irradiated MSC, supporting the notion that irradiation induced ER stress in MSC. In addition, an activator protein (GCN1) of the integrated stress response was elevated. The stress response may be induced in part by associated ROS. At proteome level, MSC responded to increased oxidative stress by elevating levels of a thiol-specific peroxidase (PRDX2) and a glutathione S-transferase (GSTP1-1).

The perception about GRP78 has changed over the past decade, as a growing number of signaling processes become apparent, which are not related to its canonical role in the ER [42,43]. It appears that GRP78 is not exclusively present in the ER but can be relocated to the cell surface (csGRP78) or even secreted into the extracellular medium (sGRP78). Both have been described to confer critical roles in the context of cancer development and cell survival [42,43]. For example, sGRP78 can act as a pro-apoptotic ligand of csGRP78 on pancreatic β-cells [44], but as a mediator of pro-survival kinase signaling in endothelial cells [45]. In addition, csGRP78 plays a mechanistic role in PI3K/AKT driven leukemogenesis [46] and in Cripto/csGRP78 regulated hematopoietic stem cell survival [47]. Therefore, monitoring of sGRP78 and targeting of csGRP78 is evaluated in anti-cancer therapy [43]. Considering these emerging roles of GRP78, non-canonical csGRP78 signaling may contribute to oncogenic signaling and impact the survival of transformed CD34+ cells. The remaining two ER proteins with increased abundance upon irradiation in MSC conditioned medium were CALR and PDIA3. In the ER, CALR participates with calnexin and PDIA3 in a process known as the calreticulin/calnexin cycle, which is involved in the folding of glycoproteins and their quality control [48]. Moreover, CALR functions as a calcium-binding lectin in calcium homeostasis and promotes MHC-I mediated antigen presentation on the cell surface [48]. In addition, mutated CALR drives JAK/STAT signaling in myeloproliferative neoplasms [49]. In the ER, PDIA3 catalyzes the rearrangement of disulfide bonds [50], thereby enabling correct folding of newly-synthesized glycoproteins [51]. Further, PDIA3 modulates STAT3 signaling from the lumen of the ER [52]. According to its catalytic activity, increased PDIA3 may alter cellular protein homeostasis. In addition, secretion of PDIA3 may activate metalloproteases and integrins in neighboring cells and thereby contribute to carcinogenesis [53]. The fact that three ER proteins with related functions were specifically increased in the conditioned medium upon MSC irradiation, while the vast majority of other cytosolic and ER proteins were unaffected, suggested a specific release rather than uncontrolled cell lysis or unspecific cellular loss of the ER. In addition, the glycolysis-related enzyme GPI, which is identical to tumor-secreted AMF, was increased in MSC conditioned medium. GPI/AMF binding to the AFM receptor results in activation of motility-mediating small Rho-like GTPases such as RhoA/Rac1 [54] and growth-mediating kinases such as MAPK/ERK [55] and PI3K/AKT [56].

In CD34+ cells, the conditioned medium from irradiated MSC induced only minute detectable changes at the proteome level after 3 days of exposure. Individual proteins participating in mitochondrial homeostasis, intracellular signaling, cytoskeleton dynamics, translation and nuclear regulation represented similar processes as in irradiated MSC. The highest increase was found for mitochondrial lon protease homolog (LONP1) in CD34+ cells. LONP1 exerts protease and chaperone activity and is therefore essential for maintaining mitochondrial protein homeostasis. Increased levels of LONP1 can be found in several cancer types [57,58,59]. In addition, proliferation-associated protein 2G4 (PA2G4) was increased in CD34+ cells, which is an activator of growth-mediating ERBB3 signaling [60]. PA2G4 is highly expressed in AML cells and stimulates cell proliferation by controlling rRNA synthesis and PCNA expression [61]. On the other hand, decreased Ras GTPase-activating-like protein IQGAP1 (IQGAP1) may promote malign development by impacting cytoskeleton dynamics, cell-cell adhesions, and signaling pathways [62]. Moreover, diminished eIF3f was detected in CD34+ cells exposed to MSC conditioned medium, which was in line with common suppression in diverse cancers [63,64]. Finally, high mobility group protein B1 (HMGB1) assumes a number of roles in cancer development as well [65]. HMGB1 enhances DNA repair and chromatin modification after DNA damage [66]. Therefore, its absence in CD34+ cells may impair genomic stability. Consequently, several modes of action, which work individually or in conjunction, may be induced by these oncogenic signals in CD34+ cells.

**Figure 4:**
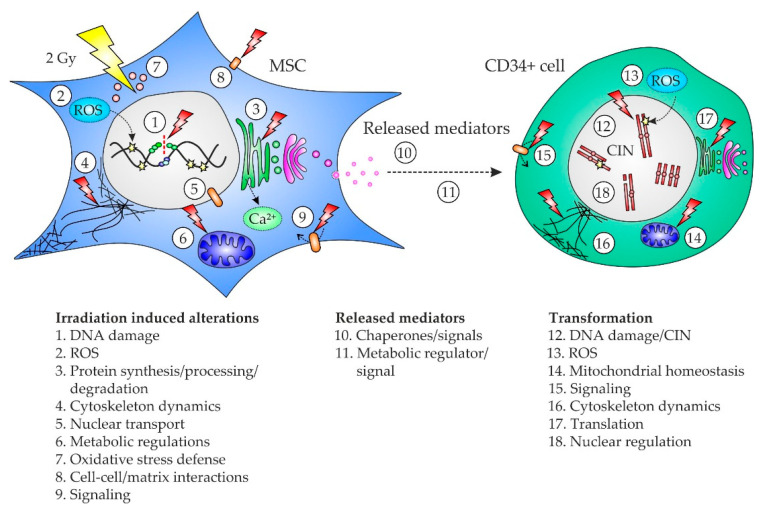
Model of the sequence of genotoxic cell-to-cell signaling from irradiated mesenchymal stromal cells (MSC) over released mediators to CD34+ cells. Irradiation of MSC induces (1) DNA damage directly and most likely indirectly by (2) reactive oxygen species (ROS). Detected protein shifts in MSC affected (3) protein synthesis/processing/degradation, (4) cytoskeleton dynamics, (5) nuclear transport, (6) metabolic regulation, (7) oxidative stress defense, (8) cell-cell/matrix interactions, and (9) intracellular signaling. Mediators released from MSC such as (10) chaperones and (11) a metabolic regulator were transmitted to CD34+ cells. In CD34+ cells, the occurrence of DNA damage and chromosomal instability (CIN) (12) was most likely mediated by increased generation of (13) ROS as well as by perturbations in (14) mitochondrial homeostasis, (15) intracellular signaling, (16) cytoskeleton dynamics, (17) translation, and (18) nuclear regulation.

Our data describe a sequence of cellular events from the primary multifaceted stress response in irradiated MSC, over transmission of genotoxic signals in conditioned medium to the induction of oncogenic mechanisms leading to DNA damage and CIN in CD34+ cells (Figure 4).

Ultimately, such genetic aberrations in CD34+ cells have the potential to stochastically initiate MN. Hence, our results provide a fundamental basis for in-depth mechanistic research and targeted therapeutic interventions to reduce NTE and the associated risk of t-MN after irradiation. Accordingly, antioxidants such as N-acetylcysteine and tempol might be able to counteract ROS in MSC and HSPC [67]. Reasoned studies are needed to address the question how the detrimental effects of IR can be ameliorated by such agents without compromising the efficacy of radiation therapy. Moreover, monoclonal antibodies such as Mab159 [68] and peptidomimetics such as BC71 [69], which target oncogenic signaling by GRP78, are candidates to reduce NTE-associated risks after irradiation.

## 4. Materials and Methods

### 4.1. Femoral Head Preparation

The femoral heads of 12 patients with coxarthrosis (7 females, 5 males, mean age: 69 years) undergoing endoprothetic surgery were collected (Table 1). The bones were broken into fragments and incubated for 1 h at 37 °C in phosphate-buffered saline (PBS) supplemented with 1 mg/mL collagenase type I (Thermo Fisher, Waltham, MA, USA). The supernatants were filtered through 100 µm pores of a cell strainer (Greiner Bio-One, Kremsmünster, Austria). MSC were grown from the fragments retained in the cell strainers in serum-free StemMACS MSC Expansion Media XF (Miltenyi Biotec, Bergisch Gladbach, Germany) supplemented with 1% penicillin/streptomycin. In line with the definition of adherence to plastic, as one of the MSC criterion by the International Society for Cellular Therapy (ISCT) [70], the adherent MSC were expanded in T175 flasks in a humidified 5% CO_2_ atmosphere at 37 °C and passaged at 80% confluency. Furthermore, CD34+ cells were enriched from the filtrates by Ficoll density gradient centrifugation and magnetic-activated cell sorting using CD34 antibody-conjugated microbeads (Miltenyi Biotec), which enables purities of about 70% [71]. CD34+ cells were grown in serum-free StemSpan SFEMII (Stemcell Technologies, Vancouver, B.C., Canada) supplemented with StemSpan Myeloid Expansion supplement (SCF, TPO, G-CSF, GM-CSF) (Stemcell Technologies) and 1% penicillin/streptomycin in a humidified 5% CO_2_ atmosphere at 37 °C.

### 4.2. Preparation of MSC Conditioned Medium

**Figure 5:**
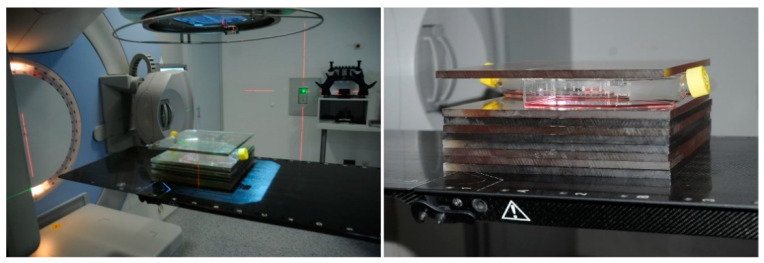
Experimental set-up for irradiation of mesenchymal stromal cells (MSC). MSC flasks were placed on 8 × 1 cm plexiglass sheets on the patient table of a Versa HD linear accelerator (Elekta). An additional 1 cm plexiglass sheet was put on top of the flasks. The laser system was used for positioning the flasks in the radiation field. All MSC in the flasks were irradiated with 2 Gy of 6 MV X-rays in orientation from top.

MSC (*n* = 12 samples from 12 patients) were grown in T175 flasks until reaching 80% confluency. MSC were rinsed in PBS before fresh serum-free StemSpan SFEMII was added. Afterwards, MSC were irradiated with 2 Gy of 6 MV X-rays in a Versa HD linear accelerator (Elekta) at room temperature and room atmosphere, while control MSC were not irradiated (Figure 5). Afterwards, the MSC were incubated in a humidified 5% CO_2_ atmosphere at 37 °C for a period of 4 h for generating MSC conditioned medium and control medium, respectively. Finally, the media were centrifuged at 4000× *g* for 10 min and supernatants were stored at −20 °C.

### 4.3. NTE Analyses

NTE were analyzed in CD34+ cells (*n* = 12 samples from 12 patients) at day 6 after culture for 3 days in untreated medium followed by culture for 3 days in conditioned medium or control medium, respectively. CD34+ cells were grown in the corresponding MSC conditioned medium of the same patient. Immunofluorescence staining of the DNA double-strand-break marker γH2AX [32] was performed using a JBW301 mouse monoclonal anti-γH2AX antibody (Merck, Darmstadt, Germany) and an Alexa Fluor 488-conjugated goat anti-mouse secondary antibody (Thermo Fisher) [72,73]. At least 50 nuclei were evaluated in each analysis. Cytogenetic analysis of G-banded chromosomes was performed according to standard procedures [74]. At least 25 metaphases were analyzed in each sample according to ISCN 2016 [75]. Cell viability was assessed using the CellTiter-Glo luminescent cell viability assay (Promega, Fitchburg, MA, USA) according to the manufacturer’s instructions. Luminescence was measured using a microplate reader (Tecan, Männedorf, Switzerland). ROS were analyzed using the ROS Detection Kit (PromoCell, Heidelberg, Germany) according to the manufacturer’s instructions, which allows the detection of hydroxyl, peroxyl and other reactive oxygen species in live cells. Luminescence was measured using a microplate reader (Tecan).

### 4.4. Protein Quantitation Using Mass Spectrometry

A proteomics approach for label-free quantitation using nanoscale liquid chromatography coupled to tandem mass spectrometry (nano LC-MS/MS) was applied for comparison of proteome differences.

### 4.5. Sample Preparation for Proteome Analysis

Samples were prepared from 2 Gy-irradiated MSC 4 h after irradiation and from non-irradiated control MSC. All MSC of 80% confluent T175 flasks were collected and washed three times in PBS. Afterwards, MSC were lysed in 200 µL RIPA buffer supplemented with Halt Protease Inhibitor Cocktail (100×) (Thermo Fisher) on ice for 30 min. Further, MSC conditioned medium and control medium were prepared using serum-free StemMACS MSC Expansion Media XF, as stated before. Finally, samples from CD34+ cells were prepared at day 6 after culture for 3 days in untreated medium followed by culture for 3 days in conditioned medium or control medium, respectively. After washing the samples three times in PBS, 1 × 10^6^ CD34+ cells of each sample were lysed in 200 µL RIPA buffer supplemented with Halt Protease Inhibitor Cocktail (100×) on ice for 30 min. Lysates were stored at −20 °C.

### 4.6. Sample Fractionation by SDS-PAGE and In-Gel Digestion

Cell culture supernatants were concentrated ten-fold before SDS-polyacrylamide gel electrophoresis (SDS-PAGE) by ultrafiltration (MWCO 5 kDa). Samples were heated to 95 °C for 5 min and cooled on ice prior to loading on NuPAGE 4–12% Bis-Tris gels (Thermo Fisher). SDS-PAGE was performed of all compared samples in parallel according to the manufacturer’s specification. Proteins were fixed within the polyacrylamide matrix by incubating the entire gel in 5% acetic acid in 1:1 (*v/v*) water:methanol for 30 min. After Coomassie staining (60 min) the gel slab was rinsed with water for 60 min. Each lane was excised and subdivided in three fractions according to protein complexity over standardized molecular weight ranges (top, middle, bottom; Tables S1 and S3). Gel fractions were cut into small pieces. Subsequently, proteins were destained by 100 mM ammonium bicarbonate/acetonitrile 1:1 (*v/v*) before reduction for 30 min in 10 mM DTT and alkylation for 30 min in 50 mM iodoacetamide. Finally, proteins were digested by trypsin overnight at 37 °C. Peptides were collected from supernatant and extracted additionally from gel pieces by 1.5% formic acid in 66% acetonitrile for 15 min. Peptides from both steps were combined and dried down in a vacuum centrifuge.

### 4.7. Mass Spectrometry

Fractions of dried peptides were re-dissolved in 35 µL 0.1% trifluoroacetic acid and analyzed individually. For this, peptides were loaded on a 75 µm × 2 cm Acclaim C18 precolumn (Thermo Fisher) using an RSLCnano HPLC system (Thermo Fisher). Then, peptides were eluted with an aqueous-organic gradient (4–44% acetonitrile, 0.1% formic acid) for 130 min and separated on a 75 µm × 15 cm Acclaim C18 column (Thermo Fisher) with a flow rate of 300 nl/min. A Triversa Automate (Advion, Ithaca, NY, USA) was used as the ion source to produce a stable electrospray, which was analyzed on a LTQ Orbitrap XL mass spectrometer (Thermo Fisher). Each scan cycle consisted of 1 FTMS full scan and up to 10 ITMS dependent MS/MS scans of the 10 most intense ions with the dynamic exclusion set to 30 sec. The mass width was set to 10 ppm and monoisotopic precursor selection was enabled. All analyses were performed in positive ion mode.

### 4.8. Comparative Proteome Analysis

Differences in proteomes between treatment groups were analyzed by Proteome Discoverer version 2.4 (Thermo Fisher). Comparisons were made between matching sample types and fractions. CD34+ cells and MSC analyses were based on five replicates. For the comparison of protein supplement-free cell culture supernatants, four replicates were utilized. The analyses were based on at least 10 ppm mass accuracy and 1% false discovery rate. Peptides were identified using the SEQUEST algorithm and a human proteome database retrieved from UniProt (https://www.uniprot.org, accessed on 15 August 2019). Protein abundance was calculated based on intensities of unique precursor ions and limited to unmodified peptides with high confidence. Precursor ion intensities were normalized to the total peptide amount in each sample. Protein abundance ratios derived from irradiated vs. non-irradiated cell samples were calculated as median of pairwise precursor comparison of replicates to reflect the pairwise experimental design of treatments. Missing intensities were imputed based on replicates, and statistics were calculated by background-based ANOVA. In cell culture supernatants, the number of required background elements was insufficient for background-based ANOVA and hence, pairwise precursor comparison was not supported. Therefore, *t*-tests were calculated for individual proteins. Furthermore, differences in cell culture supernatant were based on the top three scored unique peptides to account for protein processing such as signal peptide truncation, etc. All protein identifications were filtered for a required minimum of at least two unique peptides. A minimum of two distinct peptides with similar regulation was utilized as a requirement for calculated ratios during manual inspection. In addition, a minimum detection in at least three replicates of one group was an essential inclusion criterion for calculated ratios during manual inspection. Tables summarizing the differences in proteomes between treatment groups meet all criteria described above and include corresponding *p* values. Proteins considered to be contaminants were excluded from Table 2, but included in the Supplemental Tables S1–S3. 

### 4.9. Statistical Analysis

Proteomic data were analyzed as outlined in the section above. All other statistical calculations were done with SAS software, release 9.4 (SAS Institute, Cary, NC, USA). Wilcoxon two-sample tests were used for comparisons between the treated groups and control groups. One sample *t*-test was used in order to investigate if mean fc were different from 1.

## 5. Conclusions

In conclusion, NTE may account for a critical pathomechanism in MN initiation. Specifically, our data suggest that oncogenic signals released by irradiated MSC such as GRP78, CALR, PDIA3 and GPI/AMF are potential mediators of genetic instability in CD34+ cells. Ultimately, the identification of such mediators may define targets for the development of next-generation anti-leukemic therapies.

## Figures and Tables

**Table 1:** Non-targeted effects in CD34+ cells. fc, fold change; ISCN, international system for human cytogenetic nomenclature; NA, not assessed; Pt, patient; ROS, reactive oxygen species; [number], number of analyzed metaphases.

Pt	Age/Sex	ROS Level (fc) Irrad. MSC	ROS Level (fc) CD34+ Cells Cond. Medium	γH2AX Foci (fc) per CD34+ Cell Cond. Medium	Cytogenetics (ISCN) CD34+ Cells Control Cond. Medium		Viability (fc) CD34+ Cells Cond. Medium
#1	90/♂	NA	NA	13.0	46,XY	46,XY[20] 46,XY,chtb(5q)[1] 46,XY,chtb(10q)[1] 92,XXYY[2] 184,XXXXYYYY[1]	1.0
#2	56/♂	NA	NA	1.9	46,XY	46,XY[22] 92,XXYY[2] 184,XXXXYYYY[1]	1.0
#3	92/♀	NA	1.4	10.5	46,XX	46,XX[18] 46,XX,chtb(13q)[1] 92,XXXX[2] 184,XXXXXXXX[3] 184,XXXXXXXX, chtb(11q)[1]	0.8
#4	58/♀	NA	NA	6.9	46,XX	46,XX[19] 92,XXXX[3] 92,XXXX,chtb(6p)[1] 184,XXXXXXXX[2]	0.9
#5	85/♀	1.1	1.5	10.2	46,XX	46,XX[24] 92,XXXX,chtb(7q)[1]	1.1
#6	67/♀	1.6	NA	2.1	46,XX	46,XX[25]	1.0
#7	77/♂	1.9	NA	7.1	46,XY	46,XY[21] 47,XYY[2] 92,XXYY[1] 90,XXYY,der(1) t(1;7)x2[1]	NA
#8	54/♀	2.5	1.6	3.9	46,XX	46,XX[22] 92,XXXX[3]	1.6
#9	65/♂	1.3	NA	NA	46,XY	46,XY[19] 45,X,-Y[2] 45,X,-Y,chtb(5q)[1] 92,XXYY[2] 184,XXXXYYYY[1]	1.1
#10	58/♀	1.8	NA	6.3	46,XX	46,XX[20] 92,XXXX[2] 184,XXXXXXXX[3]	NA
#11	70/♂	1.1	1.0	NA	46,XY	46,XY[19] 92,XXYY[5] 184,XXYY[1]	1.3
#12	59/♀	2.8	0.8	NA	46,XX	46,XX[22] 92,XXXX[3]	1.1

**Table 2:** Proteome data in irradiated mesenchymal stromal cells (MSC) (*n* = 5 replicates), MSC conditioned medium (*n* = 4 replicates) and CD34+ cells grown in MSC conditioned medium (*n* = 5 replicates) in comparison to controls. Gp, group; PSMs, peptide-to-spectrum matches.

Gp	Category	Accession No.	Protein	Function	Abundance Ratio	Abundance *p* Value	Coverage	No. of Unique Peptides	PSMs
**Irradiated MSC**	Protein synthesis/ processing/ degradation	P46783	40S ribosomal protein S10 (RPS10)	40S ribosomal subunit	4.3	<0.0001	20	2	9
		O00303	Eukaryotic translation initiation factor 3 subunit F (eIF3f)	Component of eIF-3 complex	4.2	<0.0001	13	3	11
		Q10713	Mitochondrial-processing peptidase subunit alpha (PMPCA)	Subunit of essential mitochondrial processing protease	3.8	<0.0001	6	2	8
		Q92616	eIF-2-alpha kinase activator GCN1 (GCN1)	Complex with EIF2AK4/GCN2 on translating ribosomes	3.2	<0.0001	6	9	45
		P62937	Peptidyl-prolyl cis-trans isomerase A (PPIA)	Protein folding	3.1	0.0037	58	9	67
		P61289	Proteasome activator complex subunit 3 (PA28g)	Proteasome regulator	2.8	0.0101	16	3	3
		P26368	Splicing factor U2AF 65 kDa subunit (U2AF2)	pre-mRNA splicing and 3′-end processing	2.7	0.0004	20	4	17
		Q12849	G-rich sequence factor 1 (GRSF-1)	Post-transcriptional mitochondrial gene expression	2.3	0.0041	11	2	7
		Q86VP6	Cullin-associated NEDD8-dissociated protein 1 (p120 CAND1)	Key assembly factor of SCF E3 ubiquitin ligase complexes	2.3	0.0084	9	7	26
		P60842	Eukaryotic initiation factor 4A-(eIF-4A-I)	RNA helicase subunit of eIF4F complex	2.1	0.0114	39	8	63
		Q5VYK3	Proteasome adapter and scaffold protein ECM29 (ECM29)	Binds to 26S proteasome	2.1	0.0172	1	2	9
		P23381	Tryptophan-tRNA ligase, cytoplasmic (WARS1)	Aminoacylation of tRNA	2.0	0.0156	24	6	15
		Q10567	AP-1 complex subunit beta-1 (AP1B1)	Protein sorting in trans-Golgi network and/or endosomes	2.0	0.0278	14	2	57
		P61513	60S ribosomal protein L37a (RPL37A)	60S ribosomal subunit	0.39	0.0008	17	2	8
	Cytoskeleton dynamics	P07942	Laminin subunit beta-1 (LAMB1)	Component of basal membrane	2.6	0.0012	5	5	28
		O75844	CAAX prenyl protease 1 homolog (ZMPSTE24)	Cleavage of prelamin to lamin A	2.5	0.0027	5	2	4
		Q14195	Dihydropyrimidinase-related protein 3 (DRP-3)	Remodeling of cytoskeleton	2.2	0.0054	14	4	17
		Q01518	Adenylyl cyclase-associated protein 1 (CAP1)	Regulator of filament dynamics	2.2	0.0070	40	11	66
		P33176	Kinesin-1 heavy chain (KIF5B)	Microtubule-dependent motor	2.1	0.0194	4	2	4
		P08670	Vimentin (VIM)	Intermediate filaments	0.29	<0.0001	12	4	15
	Nuclear transport	O14980	Exportin-1 (Exp1)	Nuclear export of proteins and RNA	5.1	<0.0001	5	3	11
		Q96P70	Importin-9 (Imp9)	Nuclear transport receptor	4.5	<0.0001	4	2	3
		Q92621	Nuclear pore complex protein Nup205 (NUP205)	Component of nuclear pore complex (NPC)	3.4	<0.0001	4	3	7
		P55060	Exportin-2 (Exp2)	Importin-alpha re-export from nucleus to cytoplasm	2.5	0.0032	11	6	17
	Metabolic regulation	P00338	L-lactate dehydrogenase A chain (LDH-A)	Synthesizes (S)-lactate from pyruvate	3.2	<0.0001	12	3	6
		O95336	6-phosphogluconolactonase (6PGL)	Pentose phosphate pathway	2.7	0.0125	11	2	9
		Q8N0Y7	Probable phosphoglycerate mutase 4 (PGAM4)	Glycolysis	2.6	0.0191	22	4	27
	Oxidative stress defense	P32119	Peroxiredoxin-2 (PRDX2)	Thiol-specific peroxidase	qualitative	<0.0001	23	2	12
		P09211	Glutathione S-transferase P (GSTP1-1)	Conjugation of reduced glutathione	2.4	0.0340	22	3	12
	Cell-cell/matrix interactions	P04216	Thy-1 membrane glycoprotein (THY1)	Cell-cell and cell-matrix interactions, signaling (cis/trans)	3.9	0.0003	12	3	19
	Signaling	O95837/P29992	Guanine nucleotide-binding protein subunit alpha-11/14 (GNA11/14)	Activation of PLC-β: IP3 → calcium/PKC	3.8	<0.0001	6	2	8
**MSC conditioned medium**	Chaperoning/oncogenic signaling	P11021	Endoplasmic reticulum chaperone BiP (GRP78)	Unfolded protein response (UPR), endoplasmic reticulum protein degradation (ERAD) pathway	3.5	0.0227	29	13	40
		P27797	Calreticulin (CALR)	Calreticulin/calnexin cycle, calcium-binding protein	2.4	0.0036	13	4	19
		P30101	Protein disulfide-isomerase A3 (PDIA3)	Rearrangement of -S-S- bonds in proteins	2.0	0.0225	21	10	29
	Metabolic regulation/oncogenic signaling	P06744	Glucose-6-phosphate isomerase (GPI)/autocrine motility factor (AMF)	Glycolysis-related enzyme, ligand of AMF receptor	2.4	0.0006	9	4	7
**CD34+ cells**	Mitochondrial homeostasis	P36776	Lon protease homolog, mitochondrial (LONP1)	Degradation of misfolded or damaged polypeptides	4.1	<0.0001	7	2	8
	Signaling	Q9UQ80	Proliferation-associated protein 2G4 (PA2G4)	ERBB3 signaling, growth regulation, increased in AML	2.2	<0.0001	18	4	4
	Cytoskeleton dynamcis	P46940	Ras GTPase-activating-like protein IQGAP1 (IQGAP1)	Dynamics and assembly of actin cytoskeleton	0.48	<0.0001	3	2	5
	Translation	O00303	Eukaryotic translation initiation factor 3 subunit F (eIF3f)	Component of eIF-3 complex, decreased in cancers	0.40	<0.0001	8	2	12
	Nuclear regulations	P09429	High mobility group protein B1 (HMGB1)	DNA chaperone, replication, transcription, chromatin remodeling, p38-MAPK/NF-kappa B activation	0.35	<0.0001	18	3	5

## Data Availability

The data that support the findings of this study are available from the corresponding author upon reasonable request.

## Supplementary Materials

The following are available online at https://www.mdpi.com/article/10.3390/ijms22115844/s1.
